# Multimodal OCT Biomarkers of Fibrosis and Steatosis in the Regenerating Liver

**DOI:** 10.3390/ijms27146527

**Published:** 2026-07-22

**Authors:** Svetlana Rodimova, Ekaterina Gubarkova, Nikolai Bobrov, Ilya Shchechkin, Vera Kozlova, Natalia Zolotova, Pavel Bureev, Anastasia Polozova, Maria Karabut, Grigory Gelikonov, Natalia D. Gladkova, Vladimir Zagainov, Elena Zagaynova, Daria Kuznetsova

**Affiliations:** 1 Institute of Experimental Oncology and Biomedical Technologies, Privolzhsky Research Medical University, 10/1 Minin and Pozharsky Sq., 603000 Nizhny Novgorod, Russia; kgybarkova@mail.ru (E.G.);; 2 The Volga District Medical Centre of Federal Medical and Biological Agency, 14 Ilinskaya St., 603000 Nizhny Novgorod, Russia; 3 Department of Molecular Biology and Immunology, N.I. Lobachevsky Nizhny Novgorod National Research State University, 23 Gagarina Ave., 603022 Nizhny Novgorod, Russia; 4 A.V. Gaponov-Grekhov Institute of Applied Physics of the Russian Academy of Sciences, 46 Ulyanova Street, 603950 Nizhny Novgorod, Russia; 5 Nizhny Novgorod Regional Clinical Oncologic Dispensary, Delovaya St., 11/1, 603126 Nizhny Novgorod, Russia; 6 Lopukhin Federal Research and Clinical Center of Physical-Chemical Medicine of Federal Medical Biological Agency, 1a Malaya Pirogovskaya St., 119435 Moscow, Russia; 7 Laboratory of Omics and Regenerative Technologies, Institute for Regenerative Medicine, Sechenov First Moscow State Medical University (Sechenov University), 8–2 Trubetskaya St., 119991 Moscow, Russia

**Keywords:** optical coherence tomography, liver steatosis, liver fibrosis, liver regeneration, OCT elastography, stiffness, attenuation coefficient of the OCT signal, image analysis

## Abstract

The liver’s regenerative capacity is essential for resection and transplantation, but chronic liver diseases compromise this ability. Conventional preoperative tests poorly predict regeneration in diseased livers, necessitating new intraoperative tools. Optical coherence tomography (OCT) provides real-time high-resolution “optical biopsy” and functional tissue assessment, making it a promising method for evaluating liver regenerative potential. The aim of this study was to identify characteristic criteria derived from MM OCT that could be used intraoperatively to detect the presence and type of pathology, and to assess the reduction in liver regenerative potential in patients. In this study, we used intraoperative multimodal OCT (MM OCT)—combining attenuation mapping and elastography—for real-time, label-free assessment of changes in liver structure, and stiffness during regeneration. MM OCT also provides higher resolution and specificity than conventional clinical imaging. In a rat model, we induced steatosis by high-fat diet and fibrosis by CCl_4_ injections, then induced regeneration by 70% partial hepatectomy (PH). MM OCT monitoring was performed on day 0 (pre-PH), day 3, and day 7 (post-PH). Pathologies and regeneration were verified by biochemical blood tests, histological analysis, and real time-PCR for steatosis- and fibrosis-specific genes. As a result, attenuation coefficient and Young’s modulus obtained by MM OCT enable real-time assessment of liver tissue changes in steatosis and fibrosis, which vary with regeneration stage. Steatosis showed uniformly high attenuation and low stiffness before and after resection. Fibrosis exhibited heterogeneous attenuation and marked stiffness fluctuations—high pre-resection values dropped at day 3 and rose again by day 7. Attenuation coefficient detects lipid droplets in hepatocytes and identifies high-density hepatocyte zones near collagen septa, distinguishing them from densely packed unaltered cells. Thus, MM OCT identifies pathology type, lipid infiltration and collagen presence—features that serve as indicators of impaired liver regenerative capacity.

## 1. Introduction

The liver possesses a remarkable capacity to regenerate, a critical feature underpinning the success of major hepatic resections and living-donor liver transplantation. Accurately predicting the liver’s regenerative potential is paramount for surgical planning, as it directly influences postoperative outcomes and patient safety [1,2]. While extensive research has elucidated the mechanisms of regeneration in the healthy liver, in the context of chronic liver disease, the functional reserve and regenerative capacity are frequently compromised [1,2]. This creates a significant clinical challenge, as our ability to forecast postoperative liver failure or insufficient regeneration in compromised liver remains limited.

Chronic liver diseases, particularly steatosis and fibrosis, represent a growing global health burden. Metabolic dysfunction-associated steatotic liver disease (MASLD) (formerly known as non-alcoholic fatty liver disease, NAFLD), affecting an estimated 25–30% of the adult population worldwide, ranges from simple steatosis to metabolic dysfunction-associated steatohepatitis (MASH) (formerly known as non-alcoholic steatohepatitis, NASH), which can progress to fibrosis and cirrhosis. Hepatic steatosis impairs mitochondrial function, induces cellular stress, and disrupts the intricate signaling cascades required for hepatocyte proliferation [3,4]. Similarly, fibrosis and the associated architectural distortion of the liver parenchyma create a hostile microenvironment that impedes regeneration and is strongly correlated with increased complications and mortality after resection [5,6,7]. The presence of steatosis and fibrosis is a well-documented risk factor for poor surgical outcomes. However, conventional preoperative assessments, such as volumetry and liver function tests, often fall short of accurately predicting the dynamic process of regeneration in these diseased states [8,9].

In this context, there is a pressing need for novel intraoperative diagnostic tools capable of providing real-time, quantitative intraoperative assessment of liver tissue quality and its regenerative potential. MM OCT has emerged as a powerful, high-resolution, in situ imaging modality that provides a method of “optical biopsy,” offering real-time, cross-sectional visualization of tissue structure without the need for exogenous contrast agents [10,11]. Recent advancements in OCT technology have enabled not only detailed assessment of tissue architecture, but also the evaluation of dynamic functional parameters, including microvascular perfusion and the elastic properties of tissues [12,13]. This allows for advances in both diagnostic precision and real-time monitoring in medical and biological research, offering clinicians and scientists deeper insight into disease processes, treatment responses, and physiological changes at both cellular and tissue levels.

Therefore, the objective of this study was to investigate the utility of MM OCT parameters in two modalities as predictive biomarkers for liver regeneration in the presence of underlying steatosis and fibrosis. Our previous work had established proof-of-concept for using intraoperative imaging to assess regenerative capacity in healthy liver [14], demonstrating that specific structural and functional features identified using OCT do correlate with regenerative potential. However, translation of these findings to the more clinically relevant scenario of diseased liver is urgently needed. Here, we establish a clinically relevant model of regeneration in the context of pathology, with the goal of defining novel optical criteria that can enable personalized surgical decision-making and improve outcomes for patients with chronic liver disease undergoing liver resection.

## 2. Results

### 2.1. Histological Analysis

To confirm the effective induction of pathologies, standard histological analysis was performed.

**Figure 1:**
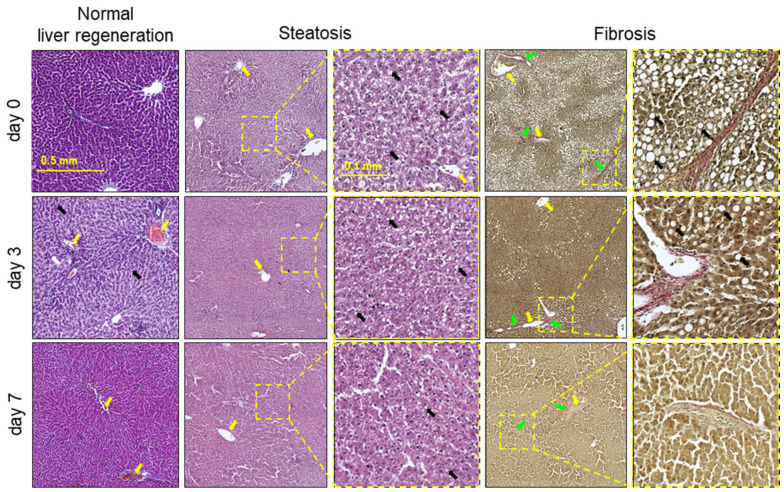
Representative histological images of liver sections (hematoxylin eosin (for normal regeneration and steatosis) and van Gieson’s stain (for fibrosis)). Arrows indicate lipid droplets (black), blood vessels (yellow) and collagen fibers (green).

In steatotic liver (on day 0), marked lipid infiltration was observed (>5% of hepatocytes containing lipid droplets), but with no evidence of nuclear displacement by the lipid droplets. Numerous hepatocytes exhibited edema (Figure 1).

In fibrotic liver (on day 0), we observed collagen accumulations forming septa, corresponding to stage F2 according to the Metavir scoring system. On day 0, and on day 3 of regeneration, pronounced hepatocyte vacuolization (a characteristic alteration in CCl4-induced toxic fibrosis) was observed, while lipid infiltration was markedly reduced by day 7 of regeneration (Figure 1).

### 2.2. Blood Biochemistry Test

**Figure 2:**
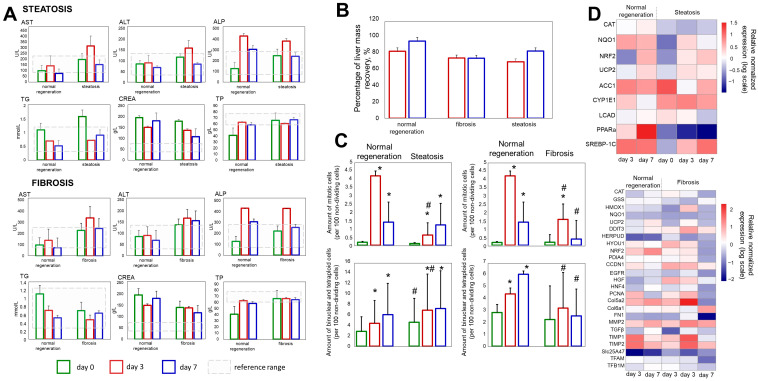
Biochemical, morphometric, and liver weight parameters during regeneration in normal, steatotic, and fibrotic liver. (**A**) Analysis of biochemical serum markers for liver function. (**B**) Liver recovery in normal state, steatosis and fibrosis models. (**C**) Morphometric analysis of the liver tissue in normal state, steatosis and fibrosis models. The charts show the number of mitotic cells and number of binucleate and tetraploid cells during regeneration. Values are presented as the number of dividing cells per 100 non-dividing cells, as means ± SD. Statistical significance (*p* ≤ 0.05) is denoted by * (vs. Day 0 control) and # (vs. normal regeneration at the corresponding time points). (**D**) Differential gene expression in liver tissue with steatosis and fibrosis during regeneration. The heat-map displays the log2 (Fold Change) in gene expression relative to healthy control liver tissue (baseline). The color key indicates gene upregulation (blue) and downregulation (red).

Biochemical parameters during normal regeneration were predominantly within typical reference ranges [15] with the following exceptions: elevated creatinine levels at all time points, increased alkaline phosphatase (ALP) on days 3 and 7, and decreased total protein on day 0 and day 7 of regeneration (Figure 2A).

In steatosis, we observed elevated levels of aspartate aminotransferase (AST), alanine aminotransferase (ALT), and ALP on day 3 of regeneration, relative to the reference values. High creatinine values were noted at all regeneration time points. On day 0, an increase in blood triglycerides (TG) levels was observed, with subsequent normalization on day 3 and day 7 (Figure 2A).

In fibrosis, we observed elevated AST and ALT levels at all regeneration time points, whereas ALP levels were increased only on day 3. Creatinine was elevated at all regeneration time points, along with high urea levels on day 0. Elevated albumin levels were detected on day 0 and day 3. TG levels remained within the reference range. However, high cholesterol levels were observed on day 0 (Figure 2A).

### 2.3. Evaluation of Liver Restoration

Assessment of liver weight recovery showed that during normal regeneration, 80.9 ± 1.1% of liver weight was restored by day 3, and 92.9 ± 3.3% by day 7. Liver weight recovery in both steatosis and fibrosis was significantly reduced compared to normal liver regeneration (Figure 2B).

Morphometric analysis of the liver tissue during normal regeneration revealed a marked increase in the number of mitotic cells on day 3 of regeneration, followed by a decrease by day 7, indicating enhanced proliferative activity of hepatocytes by day 3 of regeneration. The number of polyploid cells progressively increased from day 0 to day 7 of regeneration (Figure 2C).

In steatosis, morphometric analysis revealed that the number of mitotic cells was significantly reduced compared to normal regeneration, indicating decreased hepatocyte proliferative activity. Furthermore, the number of polyploid cells was higher than during normal regeneration, which probably indicates cell cycle arrest (Figure 2C). In fibrosis, a marked increase in the number of mitotic cells was observed on day 3 of regeneration relative to day 0. However, this proliferative activity was primarily associated with the development of pathological regenerative nodules characteristic of fibrosis. Even so, the number of mitotic cells was lower than that observed during normal regeneration. The number of polyploid cells was also reduced compared to normal regeneration (Figure 2C).

### 2.4. Molecular Analysis

In steatosis, we demonstrated decreased levels of expression of *PPAR-α* (peroxisome proliferator-activated receptor alpha), a regulator of fatty acid oxidation, and increased levels of *SREBP-1C* (sterol regulatory element-binding protein 1c) and *ACC1* (acetyl-coA carboxylase 1), regulators of lipogenesis (Figure 2D). The suppression of *PPAR-α* results in an inability of the steatotic liver to switch to lipolysis via beta oxidation for energy production, concurrently with increased expression of *ACC1* and *SREBP-1C* [16]. Consequently, following PH, the steatotic liver’s capacity to utilize fatty acids is impaired, leading to their accumulation. These accumulated fatty acids may serve as substrates for the formation of toxic compounds that promote hepatocyte damage [17]. Genes regulating the cellular response to oxidative stress exhibited lower expression in steatosis compared to normal conditions, indicating a diminished cellular protective function.

In fibrosis, increased extracellular matrix (ECM) gene expression was observed, particularly collagen *Col5a2* expression on day 3. *TIMP1* and *TIMP2* (tissue inhibitors of metalloproteinases 1 and 2) expression increased on day 3 and day 7, concomitant with increased expression of *MMP2* (matrix metallopeptidase 2), a gene encoding an enzyme that degrades the ECM (Figure 2D). Genes positively regulating proliferation (*CCND1* (cyclin D1) and *PCN*A (proliferating cell nuclear antigen)) had been activated by days 3 and 7 of regeneration; however, this was a pathological proliferation (Figure 2D). Such proliferation of hepatocytes within pathological regenerative nodules is a characteristic feature of liver fibrosis [18]. We observed low expression levels of the mitochondrial transporter *Slc25A47* (solute carrier family 25 member 47), indicating reduced mitochondrial activity and biogenesis (Figure 2D). Furthermore, a coordinated increase in the expression of ECM genes (*Col5a2* and *FN1* (fibronectin 1)) together with key markers of endoplasmic reticulum (ER) stress, notably *PDIA4* (protein disulfide isomerase family a member 4) and *DDIT*3 (DNA damage inducible transcript 3), was observed on day 3, followed by a decline by day 7 (Figure 2D). Fibrosis leads to the formation of a rigid ECM that impedes regeneration. High levels of expression of *TIMP1/2* and *MMP2* indicate active ECM remodeling, accompanied by enhanced expression of *Col5a2* and *Col6a1* genes. Suppression of genes involved in regulating mitochondrial biogenesis (*TFAM* (transcription factor A, mitochondrial) and *TFB1M* (transcription factor B1 mitochondrial)) in fibrosis is more pronounced than under normal conditions (as indicated by the more intense blue staining in Figure 2D).

### 2.5. Assessment of Structural Changes During Liver Regeneration Using OCT Attenuation Coefficient

**Figure 3:**
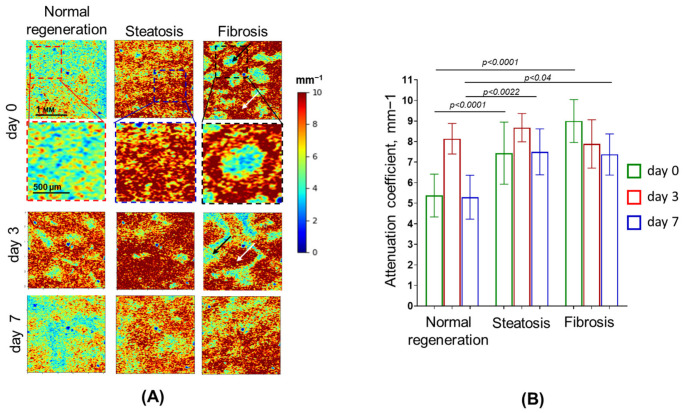
En face color-coded maps (**A**) and quantitative assessment (**B**) of the OCT attenuation coefficient of liver steatosis and fibrosis compared to normal liver during regeneration at different time points. Enlarged areas of the color-coded maps (**A**) in colored frames demonstrate normal hepatocytes (red frames), hepatocytes containing lipid droplets (blue frames), and tightly packed hepatocytes surrounded by fibrous tissue and lipid droplets (black frames). Designations: black arrows indicate hepatocytes; white arrows indicate lipid droplets. Lines denote statistically significant differences between the groups studied (Kruskal–Wallis nonparametric test with Benjamini–Hochberg correction for multiple comparisons).

In steatosis, the presence of lipid inclusions within hepatocytes led to a high level of light scattering, resulting in increased signal attenuation compared to normal liver (Figure 3A). In fibrosis, where normal hepatocytes are replaced by dense collagen fibers forming septa around tightly packed hepatocytes and numerous lipid droplets, this leads to a more heterogeneous distribution of both high (>8 mm^−1^) and low (<6 mm^−1^) attenuation coefficient values, in comparison to normal liver (Figure 3A). Quantitative comparison (Figure 3B, green boxes) revealed a statistically significant increase in attenuation coefficient values in steatosis and in fibrosis versus normal liver (7.4 ± 1.5 mm^−1^ and 9.0 ± 1.0 mm^−1^ vs. 5.3 ± 1.0 mm^−1^, respectively; *p* < 0.0001).

On day 3 of regeneration in steatosis, as in normal conditions, a further but non-significant (*p* > 0.05) increase in attenuation coefficient values was observed compared to the values before resection (day 0) (Figure 3A), probably due to the greater accumulation of small and large lipid droplets in the hepatocytes (Figure 1). In fibrosis, an opposite trend was noted on day 3 of liver regeneration—a decrease in attenuation coefficient values compared to day 0 (pre-resection); this difference was also non-significant (*p* > 0.05) (Figure 3B). Morphological analysis on day 3 of regeneration in fibrosis demonstrated a more diffuse distribution of lipid droplets among normal hepatocytes, along with collagen remodeling [19,20] (Figure 1). On day 3 of regeneration in both steatosis and fibrosis, the attenuation coefficient values were not significantly different from those observed during normal liver regeneration at the corresponding time point (Figure 3B, red boxes; *p* > 0.05).

On day 7 of regeneration in steatosis and fibrosis, a non-significant (*p* > 0.05) decrease in attenuation coefficient values (Figure 3B) and a more uniform distribution on the attenuation coefficient maps (Figure 3A) were observed compared to days 0 and 3 of regeneration. However, attenuation coefficient values for steatosis and fibrosis remained statistically significantly higher than the values at day 7 of normal regeneration (7.5 ± 1.1 mm^−1^ and 7.4 ± 1.0 mm^−1^ vs. 5.2 ± 1.0 mm^−1^, respectively; *p* < 0.05) (Figure 3B, blue boxes); this is consistent with the morphological analysis, which indicates persistent lipid infiltration and hepatocyte edema in these pathological conditions (Figure 1).

### 2.6. Assessment of Liver Stiffness Using Compression OCT Elastography

**Figure 4:**
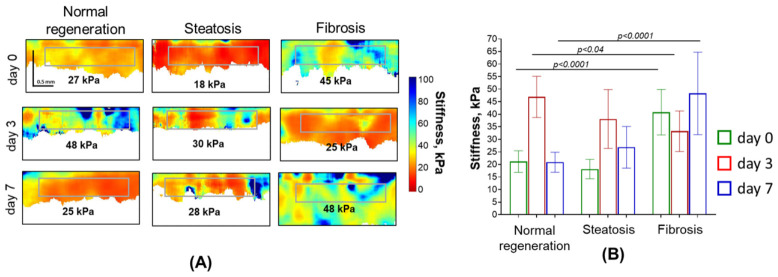
OCE images (**A**) and quantitative stiffness assessment (**B**) of rat liver in normal, steatotic, and fibrotic conditions during regeneration at various time points. In the OCE images, red indicates soft areas (<10 kPa); orange shows moderate stiffness (10–30 kPa); yellow-green marks increased stiffness (30–60 kPa); blue-cyan represents the stiffest regions (>60 kPa). Gray rectangles in (**A**) represent tissue regions for which mean stiffness values were calculated. Line segments indicate statistically significant differences between the study groups (nonparametric Kruskal–Wallis test with Benjamini–Hochberg multiple comparison correction).

In the OCT elastography (OCE) images of liver with steatosis, the homogeneously distributed low stiffness values (less than 20 kPa) differed from those observed during normal liver regeneration (Figure 4A). This is probably associated with the presence of numerous lipid droplets in the cytoplasm of the hepatocytes and with the significant cellular edema found in this pathology (Figure 1, second column). Conversely, in fibrosis, an inhomogeneous distribution of higher stiffness values (more than 40 kPa) was found compared to normal tissue (Figure 4A); this is probably related to the presence of collagen fibers forming septa around densely packed hepatocytes and to the large number of lipid droplets (Figure 1, third column). Statistically significant increases in the average liver stiffness values were observed only in the case of fibrosis compared to normal tissue (Figure 4B, 40.8 ± 9.0 kPa vs. 21.1 ± 4.2 kPa; *p* < 0.0001).

After resection, by day 3 of liver regeneration with steatosis, a heterogeneous distribution of both high and low stiffness values could be observed in the OCE images (Figure 4A), similar to that seen in normal liver tissue. There is a statistically significant increase in stiffness values on day 3 compared to the pre-resection levels (Figure 4B, 38.1 ± 11.6 kPa vs. 18.1 ± 3.8 kPa; *p* < 0.0002). This result is consistent with the morphological analysis, which, although not pronounced, demonstrated an increase in the number of proliferating hepatocytes of varying sizes and an accumulation of both small and large lipid droplets in the steatotic liver (Figure 1, second column). By contrast, in the case of fibrosis, the pattern of stiffness change after resection was opposite to that seen in normal liver and in steatosis. On day 3 of regeneration in fibrotic tissue, a statistically significant decrease in stiffness was observed compared to pre-resection levels (Figure 4B, 33.2 ± 8.1 kPa vs. 46.9 ± 8.2 kPa; *p* < 0.04). Morphological analysis showed that, by day 3 of regeneration in fibrotic liver, there was a more diffuse distribution of lipid droplets, and that collagen remodeling was underway, resulting in decreased tissue stiffness [19,20] (Figure 1, third column).

On day 7 of regeneration of steatotic liver, tissue stiffness had decreased only slightly relative to day 3 (Figure 4B; *p* > 0.05). Nonetheless, the stiffness values remained higher compared to day 7 of normal regeneration (26.8 ± 8.3 kPa vs. 20.8 ± 4.0 kPa; *p* > 0.05). These findings align with morphological analysis data, which showed no complete restoration of tissue architecture under steatosis, and both persistent lipid infiltration in cells and hepatocyte edema (Figure 1, second column). By contrast, on day 7 of fibrotic liver regeneration, stiffness values had increased again and matched the pre-resection levels. This was confirmed by evidence of the reversion of the pathological tissue structure, characterized by fibrous septa surrounding densely packed hepatocytes (Figure 1). Consequently, a significant increase in liver stiffness was observed in this case compared to the corresponding time point of normal regeneration (48.3 ± 16.3 kPa vs. 20.8 ± 4.0 kPa; *p* < 0.0001).

## 3. Discussion

In the present study, using MM OCT, we demonstrated that the attenuation coefficient and Young’s modulus values can be used for the real-time quantitative assessment of dynamic changes in steatotic and fibrotic liver tissues, and that these changes critically depend on the stage of liver regeneration.

Specifically, we observed that steatosis is characterized by uniformly high attenuation coefficient values and consistently low stiffness values at all time points before and after resection. By contrast, fibrosis exhibited a distinctly different pattern: the attenuation coefficient values were heterogeneously distributed, with clearly defined concurrent areas of either low or high values, while tissue stiffness showed dramatic fluctuations—from high pre-resection values to a sharp decrease by day 3 post-PH, followed by a subsequent increase in stiffness by day 7. We confirmed that the OCT signal attenuation coefficient can enable the detection of lipid droplets within hepatocytes and helps to identify regions of higher hepatocyte density surrounded by collagen septa—areas containing lipid droplets exhibiting a higher signal on attenuation coefficient maps than areas with a high density of unaltered hepatocytes.

Such changes are in good agreement with published data. The combination of high attenuation coefficient and low tissue stiffness in steatosis correlates well with the pronounced lipid infiltration characteristic of this condition [21,22]. It was established that in steatosis, lipid droplets within hepatocytes exhibit high degrees of light scattering, leading to an increased signal attenuation coefficient. Importantly, in contrast to normal regeneration, these high attenuation values persisted throughout all stages of regeneration—a finding that highlights a key pathological feature, that of persistent hepatocyte lipid infiltration.

Our finding that steatosis lowers liver stiffness aligns well with the known structural and compositional changes in fatty liver. Steatosis leads to intracellular lipid accumulation, which displaces normal ECM architecture and reduces the effective elastic modulus of the tissue. Although direct OCT-based stiffness measurements in steatosis have not been previously reported, our data are corroborated by independent studies using other techniques. The stiffness reduction in steatosis can be mechanistically linked to the absence of extensive collagen cross-linking, which is minimal in simple steatosis but prominent in fibrosis [19,23]. Thus, our OCT data provide in vivo validation of earlier in vitro and ex vivo observations showing that softer substrates (modulus values as low as 140–610 Pa) permit different cellular behaviors compared to stiff fibrotic matrices [23,24].

In fibrosis, low attenuation coefficient values correspond to zones of densely packed hepatocytes located within liver lobules and surrounded by fibrotic septa, whereas areas of high attenuation reflect zones of lipid infiltration. The coexistence of extensive lipid infiltration and fibrotic septa within the tissue is a hallmark of toxic fibrosis.

The changes in tissue stiffness followed an intriguing trajectory. Before resection, fibrotic liver displayed high stiffness, consistent with literature reports attributing this to the accumulation of collagen fibers. However, by day 3 of regeneration, the stiffness had dropped markedly, only to recover by day 7. This pattern is attributable to collagen remodeling following the resection. As noted in previous work, fibrosis is associated with increased matrix stiffness due to excessive collagen deposition and cross-linking by lysyl oxidases and transglutaminases [19,20]. This stiffness is detected by hepatic stellate cell (HSC) surface integrins and discoidin domain receptors, promoting HSC activation and myofibroblast differentiation [1,19,24]. Moreover, stiff matrices upregulate *TIMP-1* and downregulate *MMP-9*, creating a self-perpetuating loop that inhibits ECM degradation [20,23]. Furthermore, it has been demonstrated that collagen remodeling and HSC activation gradually decline as substrate stiffness increases beyond a certain threshold; this is consistent with our observation of stiffness re-increase after day 3, with the possible desensitization of mechanotransduction pathways [19]. This is consistent with our molecular analysis data. Fibrosis leads to the formation of a rigid ECM that impedes regeneration. The high levels of expression of *TIMP1/2* and *MMP2* that we identified indicate active ECM remodeling [25], which is accompanied by enhanced expression of the *Col5a2* and *Col6a1* genes, suggesting induction of fibrogenesis. Suppression of genes (*TFAM*, *TFB1M*) regulating mitochondrial biogenesis in fibrosis was more pronounced than under normal conditions. This is explained by the fact that fibrotic liver tissue suffers from nutrient deficiency and oxidative stress, which suppress regeneration [1]. Hyperproduction of collagen proteins inevitably leads to overload of the folding apparatus in liver cells and to activation of the unfolded protein response signaling pathways [26], manifesting as the development of ER stress [27]. Importantly, although histological analysis revealed largely unchanged fibrotic septa on day 3, molecular analyses confirmed ongoing extracellular matrix remodeling—a discordance that underscores the sensitivity of biomechanical readouts over static histology.

## 4. Materials and Methods

### 4.1. Animal Model

A series of experiments was carried out using 30 male Wistar rats with an average weight of 300–400 g. Three groups were used: (1) normal liver regeneration (day 0, n = 10; day 3, n = 5; day 7, n = 5); (2) steatosis (day 0, n = 10; day 3, n = 5; day 7, n = 5); and (3) fibrosis (day 0, n = 10; day 3, n = 5; day 7, n = 5). To induce steatosis, the laboratory animals were fed a high-fat diet [28], containing 60% of calories from fat, for 12 weeks. Induction of toxic fibrosis was carried out by the intraperitoneal injection of a 33% solution of CCl4 diluted in oil twice a week for 8 weeks [29]. The regeneration process was induced by 70% PH. After resection, each animal was placed in a clean cage and kept under the standard conditions of an SPF vivarium. To analyze the state of the liver tissue at different stages of the hepatic pathologies before the induction of liver regeneration, we examined ex vivo liver samples taken during the resection (day 0). On days 3 and 7 after resection, remnant samples (whole organs) were taken from the animals for study. Liver samples taken on days 0, 3, and 7 of normal liver regeneration served as controls.

The animals were kept, before and after the operation, in accordance with the rules adopted by the European Convention for the Protection of Vertebrate Animals; according to the National Institutes of Health Guide for the Care and Use of Laboratory Animals (NIH Publications No. 8023); and in compliance with the ARRIVE guidelines. The local ethics committee at the Privolzhsky Research Medical University (protocol №2; date: 17 February 2023) approved all the animal procedures.

### 4.2. Histological Analysis

For the histological studies, the liver was fixed in a 10% solution of buffered formalin, passed through isopropyl alcohol and embedded in paraffin. Deparaffinized 7 μm sections were stained with hematoxylin/eosin and picrofuchsin by van Gieson according to the standard protocol. For each sample, 10 micrographs were obtained (×400) using a Leica DM 2500 microscope (Munich, Germany). The degree of steatosis was assessed by the intensity of lipid infiltration of the hepatocytes, while the degree of fibrosis was assessed on the basis of characteristic morphological changes according to the Metavir scale.

### 4.3. Morphometric Analysis

The morphometric analysis was carried out using histological sections with hematoxylin and eosin and van Gieson staining. For each sample, 10 micrographs were obtained (×400) using a Leica DM 2500 microscope (Munich, Germany). Morphometric analysis was performed using the following indicators: the number of tetraploid hepatocytes (cells with brightly colored, enlarged nuclei) together with the numbers of binucleate cells and of mitotic cells. The average values of all these parameters were calculated in proportion to 100 normal cells [30]. Representative histological images of dividing hepatocytes are shown in Figure S1 in the Supplementary Materials.

### 4.4. Evaluation of Liver Weight Recovery

To determine the effectiveness of liver recovery, the liver weight was measured before the induction of regeneration and at different stages of the recovery process. The initial liver weight was calculated according to the following formula: weight of resected liver (g)/0.7. The percentage of liver weight recovery was calculated as weight of remnant (g)/initial liver weight (g) × 100—this we termed the absolute percentage of liver weight recovery, and is referred to as “absolute weight (%)”. To assess the percentage of recovery of liver tissue with induced pathologies relative to the corresponding day of normal regeneration, we calculated the relative percentage of liver weight recovery, i.e., the “relative weight (%)”. For this purpose, the values of “absolute weight” for normal liver regeneration were taken as 100% (absolute weight_norm_) and divided into “absolute weight” for the liver with pathology (absolute weight_path_) at the corresponding time point of regeneration, i.e., day 3 or day 7 after PH: absolute weight_path_(g)/absolute weight_norm_(g) × 100%.

### 4.5. Biochemical Blood Tests

Blood samples were collected from the tail vein of each rat at all monitoring time points. These samples were centrifuged at 300× *g* for 15 min to separate the serum, and then the concentrations of relevant materials in the serum samples were determined. Levels of AST, ALT, ALP, TP, urea, creatinine (Crea), and TG were recorded. The analysis was carried out using a semi-automatic biochemistry/coagulation analyzer (Dymind DP-C16, Shenzhen, China) and standard reagents (Diakon-DS, Saint Petersburg, Russia) in accordance with the manufacturer’s protocols.

### 4.6. Multimodal OCT and Data Analysis

This study used a common path spectral domain multimodal OCT device (Institute of Applied Physics of the Russian Academy of Sciences, Nizhny Novgorod, Russia) with a central wavelength of 1310 nm, an axial resolution of 15 µm, a lateral resolution of 20 μm, and a scanning speed of 20,000 A-scans per second [31]. The OCT system acquired both 3D blocks of OCT data (2 mm in depth in air over a 2.4 × 2.4 mm^2^ area) and 2D lateral scans with a similar field of view. Each acquisition took 26 s. Two types of images—structural OCT and C-OCE images—could therefore be acquired simultaneously in real time from one tissue area. A more detailed description of the experimental setup and OCT data acquisition can be found in our recent publication on normal liver regeneration in rats [14].

To analyze any structural changes during liver regeneration, the OCT signal attenuation coefficient was calculated. For this we employed the depth-resolved method proposed by K.A. Vermeer [32] and modified by A. Moiseev [33]. Attenuation maps in en face projection were generated as 2D color-coded images. The attenuation coefficient was computed from approximately 50 µm to 600 µm of tissue depth, which allowed detailed visualization of the liver morphology [14].

The liver tissue stiffness was studied using compression OCE based on phase-sensitive strain visualization [34]. The axial interframe strain was estimated using the “vector” method [35], which was further improved to provide enhanced image quality under noisy conditions [36,37]. A silicone reference layer with a calibration Young’s modulus of 40 kPa was placed between the OCT probe and the tissue surface. Gentle compression was applied, so the processed images mapped the strains, allowing calculation of the tangent elastic Young’s modulus (kPa) at a standardized stress level (2 kPa). The 2D OCE maps, with a resolution of about 40–50 µm, displayed stiffer areas in blue and softer areas in red.

### 4.7. Real-Time Quantitative PCR

Total RNA was isolated using the HiPure Total RNA kit (Magen, Guangzhou, China) according to the manufacturer’s protocol. Following DNase treatment and purification, cDNA was synthesized using the RNA Scribe RT Minus Kit (Biolabmix, Novosibirsk, Russia). qPCR was performed using a reaction mix containing SYBR Green I (Invitrogen, Carlsbad, CA, USA) on a Bio-Rad FX96 Real-Time PCR System (Bio-Rad, Hercules, CA, USA) under standard thermal cycling conditions. Reaction efficiency for each assay was determined using a standard curve. The *Hprt1* and *Ywhaz* genes were used as reference genes. Quantitative analysis was performed using CFX Maestro 2.3 software.

### 4.8. Statistical Analysis

All statistical analyses were performed using the R programming language. For all tests, a *p*-value ≤ 0.05 was considered statistically significant.

To assess differences in liver weight recovery between groups, the non-parametric Kruskal–Wallis test was applied with a Dunn’s post hoc test, comparing data at each time point against the corresponding normal regeneration control. Statistical significance for morphometric analysis was determined using a one-way analysis of variance (ANOVA) followed by Tukey’s post hoc test for multiple comparisons.

Statistical analysis was carried out for the morphometric analysis data, the blood biochemical analysis and the data on attenuation coefficient, as well as for vessel density and stiffness as calculated from the 3D OCT, OCTA and OCE images, respectively. The normality of the data distribution was assessed using the Shapiro–Wilk test. For the morphometric analysis data, the normality of data distribution was confirmed. For biochemical blood tests and the 3D OCT, OCTA and OCE data, it was shown that normal distribution was not characteristic of all data sets. In this regard, the Mann–Whitney U-test was chosen to compare these data sets. For each studied parameter, three time points were analyzed, and three paired comparisons were performed. To reduce the possibility of a type I error caused by multiple comparisons, the obtained *p*-value was multiplied by 3 (the number of comparisons) based on the Bonferroni multiple comparison correction method. In all cases, the differences were considered statistically significant when *p* < 0.05. Diagrams were generated using Prism 8.0.2 statistical software (GraphPad Software, San Diego, CA, USA).

## 5. Conclusions

In this study, multimodal OCT enabled the identification of liver pathological features such as lipid infiltration and collagen deposition based on an assessment of the resulting changes in light-scattering and elastic properties. The obtained OCT data were comprehensively verified based on blood biochemical markers, observation of liver tissue histology, and changes in the expression of steatosis- and fibrosis-specific genes. The obtained data provide a method for detecting violations of the liver’s regenerative capacity. We have demonstrated that both the attenuation coefficient and tissue stiffness of liver tissue exhibit pathology-specific and regeneration-stage-dependent dynamics during steatosis and fibrosis. Steatosis is characterized by persistently high attenuation coefficient values and uniformly low tissue stiffness throughout all phases of liver regeneration, reflecting stable lipid droplet accumulation and the absence of extensive collagen cross-linking. In contrast, fibrosis presents a heterogeneous attenuation pattern with alternating low- and high-value zones, alongside intriguing trends in stiffness change—high stiffness before resection, with a sharp decline by Day 3 of regeneration, and a subsequent rise by Day 7—indicative of active collagen remodeling and matrix reorganization. Importantly, the biomechanical readouts captured dynamic changes that were not evident from histology alone, underscoring the added value of OCT-based tissue characterization. Collectively, these results confirm that multimodal OCT is a powerful tool for differentiating pathologies, assessing the degree of lipid infiltration, and detecting collagen deposits based on the light-scattering and elastic properties of the hepatic tissues, which can predict a decline in liver regenerative capacity.

## Data Availability

The original contributions presented in this study are included in the article. Further inquiries can be directed to the corresponding author.

## Supplementary Materials

The following supporting information can be downloaded at: https://www.mdpi.com/article/10.3390/ijms27146527/s1.

## Abbreviations

The following abbreviations are used in this manuscript:

OCT	Optical Coherence Tomography
MM OCT	Multimodal Optical Coherence Tomography
PH	Partial Hepatectomy
NAFLD	Non-Alcoholic Fatty Liver Disease
NASH	Non-Alcoholic Steatohepatitis
ALP	Alkaline Phosphatase
AST	Aspartate Aminotransferase
TG	Triglycerides
PPAR-α	Peroxisome Proliferator-Activated Receptor alpha
SREBP-1c	Sterol Regulatory Element-Binding Protein 1c
ACC1	Acetyl-coA Carboxylase 1
ECM	Extracellular Matrix
TIMP1	Tissue Inhibitor of Metalloproteinases 1
TIMP2	Tissue Inhibitor of Metalloproteinases 2
MMP2	Matrix Metallopeptidase 2
Slc25A47	Solute Carrier Family 25 Member 47
FN1	Fibronectin 1
PDIA4	Protein Disulfide Isomerase Family a Member 4
DDIT3	DNA Damage Inducible Transcript 3
TFAM	Transcription Factor A, Mitochondrial
TFB1M	Transcription Factor B1 Mitochondrial
OCE	OCT elastography
