# Supplementary material for: Multimodal OCT Biomarkers of Fibrosis and Steatosis in the Regenerating Liver
Source: Int J Mol Sci. 2026 Jul 22;27(14):6527. doi: 10.3390/ijms27146527 (PMC13411441; doi:10.3390/ijms27146527)
Supplement: Supplementary file 1 [file ijms-27-06527-s001.zip › ijms-4430759-supplementary.pdf]

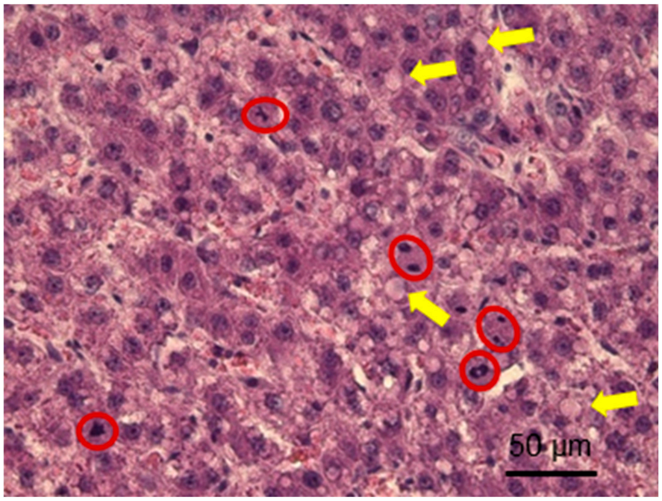

Figure S1. Representative histology image of liver tissue during regeneration. Mitotic hepatocytes are outlined in red. Yellow arrows indicate lipid droplets. Scale bar 50 µm.
